# Adipose triglyceride lipase: the first transacylase for FAHFAs

**DOI:** 10.1093/lifemeta/loac016

**Published:** 2022-08-12

**Authors:** Juan Wang, Guosheng Liang, Tong-Jin Zhao

**Affiliations:** State Key Laboratory of Genetic Engineering, Shanghai Key Laboratory of Metabolic Remodeling and Health, Institute of Metabolism and Integrative Biology, Zhongshan Hospital, Fudan University, Shanghai 200438, China; Department of Molecular Genetics and Center for Human Nutrition, University of Texas Southwestern Medical Center, Dallas, TX 75390, United States; State Key Laboratory of Genetic Engineering, Shanghai Key Laboratory of Metabolic Remodeling and Health, Institute of Metabolism and Integrative Biology, Zhongshan Hospital, Fudan University, Shanghai 200438, China

**In a recent article published in *Nature*, Patel *et al.* identified adipose triglyceride lipase (ATGL, also known as patatin-like phospholipase domain containing 2) as the first biosynthetic enzyme of fatty acid esters of hydroxy fatty acids (FAHFAs), further expanding the knowledge on bioactive lipid research and being a potential paradigm shift for ATGL studies.**

FAHFAs are a class of diverse lipids that contain two fatty acids linked by a hydroxyl ester group [1]. Although FAHFAs were initially identified in insects and plants, the intense interest was not ignited until 2014 when a landmark study by Yore *et al*. [2], discovered a subfamily of FAHFAs with anti-inflammatory and anti-diabetic properties. To date, more than 50 families (defined by the difference in acyl chains) with over 600 regioisomers (classified by the position of the ester bond linking the acyl chains) of FAHFAs have been identified [1]. Elucidation of the physiological role and regulation of endogenous FAHFAs, however, has been hindered by several challenges. One is the technical difficulty to precisely detect and quantify the low (nanomolar range) level of specific FAHFA species among the diverse families and regioisomers without the potential interference from artifacts of FA dimmers [2–4]. The other is the incomplete understanding of the synthesis and degradation of endogenous FAHFAs *in vivo*. Four putative FAHFA hydrolases, carboxyl ester lipase, androgen-dependent tissue factor pathway inhibitor regulating protein, androgen-induced gene 1, and hormone-sensitive lipase (HSL), have been identified and shown to cleave the FAHFA ester bond *in vitro*. To better understand the dynamics of FAHFAs, identification of the biosynthetic enzyme for FAHFA is in urgent need [1].

A major breakthrough was reported recently in which Kahn and colleagues identified ATGL as the first enzyme that can catalyze the formation of ester bond between FA and HFA to synthesize FAHFA [5]. This work is built on their previous studies that showed an 8- to 16-fold elevation of FAHFA levels in white adipose tissue of adipose-specific Glut4 overexpression (AG4OX) mice [2]. The authors reasoned that the dramatic increase of FAHFAs is at least partially due to elevated FAHFA synthesis in AG4OX adipose tissue, thereby making it a good model system to identify FAHFA biosynthetic enzymes. As a first step, the authors set up an FAHFA biosynthetic assay system in adipocytes by adding exogenous 9-hydroxy stearic acid (9-HSA) and Cis-10-heptadecenoic acid (C17:1) and measuring the formation of 9-C17:1-HSA. C17:1 FA is chosen because of its low-abundance endogenous level, thus avoiding the inferences from large background signals. Using this assay system, AG4OX adipocytes exhibited 2-fold increase of 9-C17:1-HSA compared to WT adipocytes. To make this assay more robust, the authors tried to block the degradation of FAHFA by treating cells with methyl arachidonyl fluorophosphonate (MAFP), a covalent inhibitor of serine/threonine lipase that inhibits all four known FAHFA lipases. Instead of the anticipated increase of FAHFA after MAFP treatment, the authors noticed a dose-dependent decrease of FAHFA, suggesting that the FAHFA biosynthetic enzyme(s) are also inhibited by MAFP. Taking advantage of this surprising finding, the authors carried out activity-based protein profiling with a fluorophosphonate (FP)-alkyne probe, which has the same functional group as MAFP and exhibites the same inhibitory activity of FAHFA synthesis [5]. Following covalent labeling with the FP-alkyne probe, candidate proteins (FP-alkyne targets) were biotinylated by click chemistry, enriched by streptavidin pull down, and analyzed by proteomics. Among the several candidates identified, only ATGL passed all the *in vitro* and *in vivo* analyses to be a FAFHA biosynthetic enzyme. Interestingly, ATGL does not synthesize FAHFA directly from FAs and HFAs. Instead, ATGL utilizes triglyceride (TG) and to a less extent diglyceride (DG) as donors of FA, and transfers it to HFA to generate FAFHA. It is worth noting that previous *in vitro* studies have shown that ATGL can carry out acyl-CoA-independent transacylase reaction using monoglyceride (MG) and DG as acyl donors and DG as acyl acceptors to synthesize TG or to remodel TG with alternative acyl compositions [6–8]. The current study [5], however, is the first to demonstrate a direct role of ATGL transacylase activity in transferring the donor FA (from TG to DG) to HFA for the endogenous FAHFA biosynthesis in adipocytes. As previous studies are mainly focusing on the TG hydrolase activity of ATGL, the transacylase activity might be a paradigm shift, especially when considering the potentially important physiological and pathophysiological roles of the bioactive FAHFAs.

**Figure 1:**
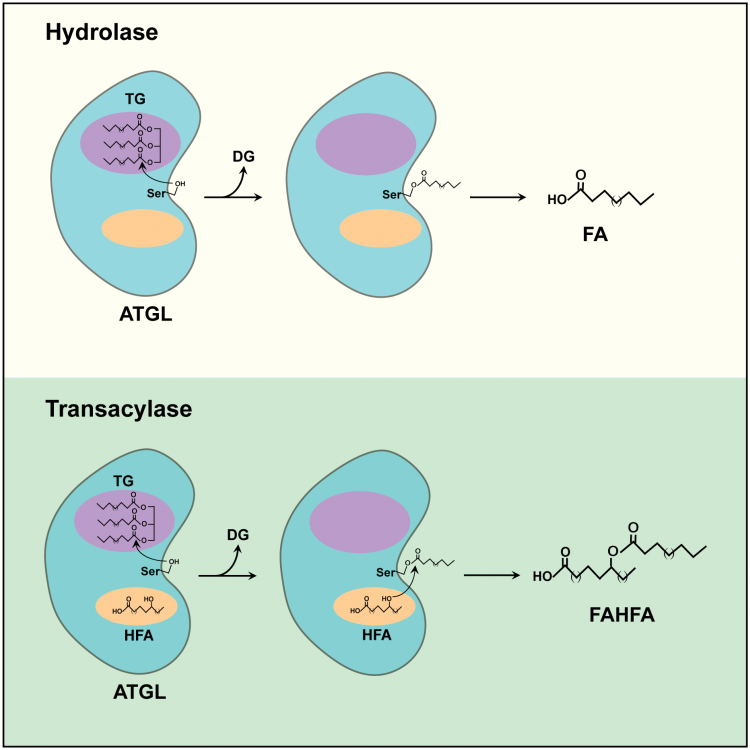
ATGL possesses hydrolase and transacylase activities. ATGL has two binding pockets, one for TG and the other for hydroxy fatty acid (HFA). When only TG binds to its pocket, ATGL functions as a hydrolase to break TG into DG and FA. When both pockets are occupied, ATGL functions as a transacylase by first hydrolyzing the TG molecule and then transferring FA to HFA to form FAHFA.

This study also raises several important questions. An immediate one is whether this transacylase activity of ATGL is distinctly or coordinately regulated by its extensively characterized lipase activity. ATGL, together with co-activator CGI-58, has been well established as a lipase that carries out the first step of TG hydrolysis to produce DG and FA [9]. Subsequent hydrolytic steps are mediated by HSL (DG hydrolase) and monoglyceride lipase (MGL, MG hydrolase). The current study [5] indicates that the transacylase and lipase activities of ATGL are intimately intertwined. Both activities require co-activator CGI-58 and exhibit much higher substrate specificity for TG than for DG. Furthermore, ATGL transacylase activity (for FAHFA biosynthesis) is abolished when the ATGL lipase activity is blocked by chemical inhibitor atglistatin or inactivated by point mutation. Therefore, the hydrolase and transacylase activities might not be separable. For both activities, the FA molecule will first need to be hydrolyzed from TG and likely forms the same acyl FA-enzyme intermediate. The only difference is whether acyl FA is transferred to water (lipase) or to HFA (transacylase) (Fig. 1). Thus, the availability of HFA might be the key to determine whether ATGL functions as a hydrolase or a transacylase. The source of HFA and how HFA is delivered to ATGL remain to be answered. It would also be important to elucidate the biochemical mechanism by which HFA and/or other factors regulate the switch of ATGL between lipase and transacylase activities. In this regard, it would be worthwhile to determine whether it is possible to use alanine-scanning to identify mutations in ATGL and/or CGI-58 that affects only lipase or transacylase activity. Of course, a crystal or Cryo-EM structure of ATGL in complex with TG and/or HFA will certainly provide structural insights into the two ATGL enzymatic activities.
